# Stroke care in the United Kingdom before, during, and after the COVID-19 lockdowns: A retrospective nationwide cohort study

**DOI:** 10.1371/journal.pone.0330903

**Published:** 2025-09-02

**Authors:** Youssef Hbid, Kaili Stanley, Charles D. A. Wolfe, Ajay Bhalla, Martin James, Abdel Douiri, on behalf of the SSNAP Collaboration

**Affiliations:** 1 School of Life Course and Population Health Sciences, King’s College London, Guy’s Campus Great Maze Pond, London; 2 The Sentinel Stroke National Audit Programme, King’s College London, Guy’s Campus Great Maze Pond, London; 3 Guy’s and St Thomas’ NHS Foundation Trust, London; 4 Royal Devon and Exeter Hospital and University of Exeter Medical School, Exeter; Azienda Ospedaliero Universitaria Careggi, ITALY

## Abstract

**Background and aim:**

The COVID-19 pandemic imposed significant pressures on healthcare services such as stroke care. This study aimed to assess the quality of stroke care and, outcome before, during and post lockdown.

**Methods:**

Nationwide registry-based cohort study of patients with acute stroke admitted to hospitals in England, Wales, and Northern Ireland were analyzed. This included 114 hospitals for study cohorts of 261,451 in the pre-pandemic control period (01/04/2017–25/03/2020). The exposures studied include 16,843 during the first lockdown (26/03/2020–23/06/2020), 48,004 during the second lockdown (05/11/2020-17/05/2021), and 82,732 post-lockdowns (18/07/2021–30/06/2022).

Logistic regression was used to compare odds of receiving aspects of acute stroke care across pandemic periods compared to the pre-pandemic period. Survival after stroke was assessed using restricted mean survival time (RMST) analysis, with models adjusted for age, sex, and stroke severity.

**Results:**

Admission to a stroke unit within 4-hours increased by 8% during the first lockdown but fell by 7% in the second lockdown and remained lower post-pandemic. During the first lockdown, brain imaging within 1-hour increased by 3%, but was not maintained thereafter. Stroke multidisciplinary access increased during the first lockdown but decreased in subsequent periods. Access to thrombectomy sequentially increased across time periods; by 40% during first lockdown (adjusted Odd-Ratio: 1.4; 95% CI:1.24–1.58), second lockdown (aOR: 1.77; 1.66–1.92), and 2-folds post-lockdown (aOR: 2.03; 1.92–2.15). Thrombolysis rates fell during the pandemic and did not recover post-pandemic. Although proportion of patients discharged with good recovery did not alter, 7-day mortality increased by 10% (hazard ratio: 1.10, 1.04-1.18) in the first lockdown period but improved thereafter.

**Conclusion:**

This nationwide population data showed unprecedented levels of pressure from the COVID-19 pandemic which have had an enduring effect on the quality of hospital stroke care and patient outcomes. Stroke care has not fully recovered post-pandemic period suggesting limited resilience.

## Introduction

The coronavirus disease (COVID-19) pandemic led to major disruption in the delivery of stroke services globally, with evidence of delayed and reduced admissions to hospital and variation in the delivery of hyperacute interventions [1]. A global review of 369 studies relating to COVID-19 showed that 47% of acute neurological care services and 63% of cross-sectoral services were disrupted, consequent on travel restrictions due to lockdowns (82%), and regulatory closure or modification of services (65%) [2]. In the United Kingdom, where the first wave of the pandemic arrived during March-April 2020, there was a substantial and abrupt fall in hospital attendance with acute stroke [3], with up to a third of stroke patients between 1 January and 31 October 2020 remaining at home [4]. Reperfusion rates fell by over 25% during the first wave of the pandemic in early 2020 in China [5], and similar falls were reported in French centers, associated with increased delays in imaging and groin puncture [6]. Other countries reported less pronounced variations in the quality of stroke care, potentially reflecting more organized healthcare and less restrictive lockdown policies in those centers [7]. It has previously shown that although stroke admissions fell sharply during the initial wave of the pandemic, key processes of stroke care improved, likely because of reduced demand and enhanced capacity within stroke services [3]. Whilst many stroke centers have reported their experience and the consequences of the pandemic during the first wave in early 2020, there is more limited data reported across the entire duration of several ‘waves’ of the pandemic or extending into the post-lockdown ‘recovery’ period. Learning from the diverse impacts of COVID-19 on health services will for the first time enable us to understand the effects of a large public health emergency on the health system and adapt plans to prevent failures of service in the future.

The aim of this study is to evaluate the quality of stroke care and outcomes between 2017–2022 covering the pre-pandemic, first and second nationwide lockdowns, and subsequent post-pandemic period using the nationwide stroke registry for England, Wales and Northern Ireland.

## Methods

### Ethical considerations

Permission for SSNAP to collect patient data without explicit consent was granted by the Confidentiality Advisory Group of the Health Research Authority under Section 251 approval.

### Data source

Data were collected by the Sentinel Stroke National Audit Programme (SSNAP), a national quality improvement registry for stroke care, which includes all hospitals admitting patients with acute stroke in England, Wales and Northern Ireland (covering 92% of the population of the UK). SSNAP collects data on all adult patients (age ≥ 18 years) admitted to hospital with acute stroke (ischemic, intracerebral hemorrhage or undetermined type). The index date is the date of admission for patients with stroke onset outside of hospital or the date of stroke onset for patients having an acute stroke whilst an inpatient (typically 5% of the total). Data are submitted prospectively on all patients presenting with acute stroke by clinical teams using a secure website from the time of admission up to 6 months after stroke, and include data on demographic and clinical characteristics, treatments and care processes, and outcomes. Overall case ascertainment of SSNAP pre-COVID-19 by comparison with administrative datasets is estimated to be 95% of all acute stroke admissions. (Data is available through a formal data access application process to SSNAP and the Healthcare Quality Improvement Partnership, which is the data controller.

### Study design

To control the spread of COVID-19 infection, severe population-level viral transmission preventive measures were introduced in the UK between 26/03/2020–23/06/2020 (“first lockdown”), and 05/11/2020-17/05/2021 (“second lockdown”), mandating social distancing measures and the closure of most schools, workplaces, retail, and recreational facilities. Acute hospital sites were included in the study if they continued to prospectively submit data to SSNAP during the period 2017–2022. Specifically, patients were eligible for inclusion in the study if their index date was during the first or second lockdown or the post-lockdown periods or were a date-matched control (exposures) during the equivalent periods in 2017, 2018 or 2019 (to correct for seasonal effects). In total, 114 hospitals provided data for a study cohort of n = 261,451 in the pre-COVID period, n = 16,843 during the first lockdown, n = 48,004 in the second lockdown, and n = 82,732 in the post-lockdown period (18/07/2021–30/06/2022). Average admissions during the corresponding matched control periods in 2017–2019 were 21,388, 45,702, and 84,701 respectively.

### Statistical analysis

Descriptive statistics were reported for patient demographics (age at time of stroke, sex, ethnicity), comorbidities (atrial fibrillation, diabetes, hypertension, congestive heart failure and previous stroke or transient ischemic attack), clinical characteristics (stroke type, onset in or out of hospital, time from onset to admission, pre-stroke disability (modified Rankin Scale score [mRS]), stroke severity (National Institutes of Health Stroke Scale [NIHSS] score), interventions and care quality metrics (stroke unit admission within 4 hours of arrival at hospital, brain imaging within 1 hour, intravenous thrombolysis administration and door to needle time, mechanical thrombectomy rate, swallow screening, time to stroke specialist nursing and physician review, therapy assessments) and outcomes (case fatality within 7 and 30 days of admission, disability at discharge from hospital [mRS]). Comparisons between categorical variables were performed using chi-squared tests. Logistic regression was used to compare odds of receiving aspects of acute stroke care across pandemic periods, with the pre-pandemic period as reference. Changes over time in case fatality were analyzed. Because the proportionality of the hazards assumption was violated, we assessed survival time after stroke by comparing the restricted mean survival time (RMST) between groups [8].

We reported survival estimates and differences in restricted mean survival time (RMST) between groups. Where noted, hazard ratios were derived from pseudo-value–based regression models of the RMST, and were adjusted for age, sex, and stroke severity.

All statistical analyses and graphical presentations were performed using R version 3.6.3.

## Results

A total of 409,030 patients with confirmed acute stroke were admitted during the equivalent periods across the 5 consecutive years of the study (2017–2022). During the 3-year pre-COVID period, SSNAP recorded an average of 240 stroke admissions per day, which fell by 21% during the first lockdown. However, admissions during the second lockdown were higher by 4% and then returned to pre-pandemic levels during the post-lockdown period. Patients admitted during the lockdown periods were broadly similar to historical controls, but there were modest differences in some patient characteristics (Table 1). There was a lower proportion of patients aged ≥80 years and fewer patients from white backgrounds during and post-pandemic. We observed a longer time from onset to hospital arrival and higher proportions of patients presented with diabetes during the lockdown and post pandemic periods (Table 1). Post-pandemic, there were lower rates of ambulance conveyance to hospital, and a corresponding increase in self-presenters. There was a shift in the distribution of pre-stroke functioning with a lower proportion of patients with severe pre-stroke disability (mRS = 5) admitted during the first lockdown and higher NIHSS on arrival during the first lockdown (Table 1).

**Table 1:** Patient characteristics.

	Pre-pandemic	First lockdown	Second lockdown	Post pandemic
**Dates**	01/04/2017–25/03/2020	26/03/2020–23/06/2020	05/11/2020–17/05/2021	18/07/2021–30/06/2022
**n**	261451	16843	48004	82732
**Average admissions per day**	240	189	249	238
**Sex (female)**	125932 (48%)	7888 (47%)	22592 (47%)	38632 (47%)
**Age on arrival, median [IQR]**	77.0 [66; 85]	76.0 [65; 84]	76.0 [65; 84]	76.0 [65; 84]
**Age groups**				
**≤ 59 years**	38641 (15%)	2713 (16%)	7627 (16%)	13166 (16%)
**60-69 years**	41958 (16%)	2736 (16%)	8160 (17%)	13908 (17%)
**70-79 years**	71511 (27%)	4781 (28%)	13312 (28%)	23189 (28%)
**80-89 years**	81166 (31%)	5019 (30%)	14249 (30%)	24174 (29%)
**> 90 years**	28075 (11%)	1594 (9%)	4656 (10%)	8295 (10%)
**Ethnicity**				
**White**	229581 (88%)	14394 (85%)	41019 (85%)	70140 (85%)
**Asian**	8487 (3%)	569 (3%)	1761 (4%)	3155 (4%)
**Black**	3820 (1%)	216 (1%)	917 (2%)	1466 (2%)
**Mixed**	1017 (0.4%)	63 (0.4%)	188 (0.4%)	358 (0.4%)
**Other**	3476 (1%)	184 (1%)	606 (1%)	1358 (2%)
**Ethnicity unknown**	14970 (6%)	1417 (8%)	3513 (7%)	6255 (8%)
**Comorbidities**				
**Atrial Fibrillation**	50170 (19%)	3059 (18%)	8731 (18%)	15272 (18%)
**Diabetes**	57486 (22%)	3878 (23%)	11009 (23%)	19501 (24%)
**Hypertension**	143029 (55%)	9402 (56%)	26890 (56%)	45896 (55%)
**Congestive Heart Failure**	13684 (5%)	920 (5%)	2578 (5%)	4597 (6%)
**Previous Stroke or transient ischemic attack**	67511 (26%)	4183 (25%)	11704 (24%)	19796 (24%)
**Pre-stroke disability (mRS)**				
**0**	136085 (52%)	882 (52%)	24704 (51%)	41056 (50%)
**1**	43169 (17%)	2909 (17%)	8372 (17%)	15078 (18%)
**2**	28730 (11%)	1885 (11%)	5380 (11%)	9453 (11%)
**3**	32198 (12%)	2024 (12%)	6034 (13%)	10642 (13%)
**4**	16545 (6%)	977 (6%)	2781 (6%)	5118 (6%)
**5**	4624 (2%)	227 (1%)	733 (2%)	1385 (2%)
**Arrival method**				
**Ambulance**	195027 (75%)	13130 (78%)	36833 (77%)	57803 (70%)
**In-hospital stroke**	15089 (6%)	789 (5%)	2480 (5%)	4279 (5%)
**Self-presented**	51235 (20%)	2924 (17%)	8691 (18%)	20650 (25%)
**Symptom onset to arrival (minutes), median [IQR]**	486 [127; 1338]	513 [131;1376}	526 [138;1424]	555 [156;1454]
**Stroke type**				
**Ischemic**	227787 (88%)	14677 (88%)	41571 (87%)	72102 (87%)
**Hemorrhagic**	32395 (12%)	2094 (12%)	6285 (13%)	10362 (13%)
**Stroke severity on arrival (NIHSS score)**				
**NIHSS on arrival, median [IQR]**	4 [2; 11}	5 [2; 11]	4 [2; 10}	4 [2; 10}

Compared to the pre-pandemic control period, there was an 8% absolute increase (ABI 8%; P < 0.001) in the first lockdown for direct admission to a stroke unit within 4 hours of hospital arrival. However, this fell significantly in the second lockdown and remained lower in the post-pandemic period (ABI −7% and ABI −15% respectively, p < 0.001).

The proportion of patients treated with intravenous thrombolysis was unchanged during the first lockdown but fell significantly in both the second lockdown and post-pandemic periods (ABI −1.2%, p < 0.001).

Compared to the pre-pandemic period, patients had 40% increased odds (adjusted OR: 1.4; 95% CI:1.24–1.58) of receiving thrombectomy during the first lockdown, 77% increased odds (aOR: 1.77; 95% CI:1.66–1.92) during the second lockdown, and 2-fold increased odds (aOR: 2.03; 95% CI:1.92–2.15) in the post-pandemic period.

We observed significant falls in the proportions of direct admission to a stroke unit during the second lockdown and post-pandemic periods (Table 2). During and post lockdown, stroke specialist physician assessment within 24 hours of arrival increased, by an average of 20% (p < 0.001) compared to pre-pandemic, but no significant changes were observed in stroke specialist nurse assessment within 24 hours. Delays in assessments were observed in physiotherapy, occupational, and speech and language therapy assessments within 72 hours during the second lockdown period which deteriorated further in the post-pandemic, although the latter was partially offset by a higher proportion of patients being considered ineligible for therapy (Table 2).

**Table 2:** Care quality.

	Pre-pandemic	First lockdown	Second lockdown	Post Pandemic
**First ward of admission**				
** Stroke Unit**	207340 (79%)	13451 (80%)	36867 (77%)	62570 (76%)
** Acute Medical Admissions Unit**	34570 (13%)	1795 (11%)	6753 (14%)	11479 (14%)
** Critical Care/Intensive Care Unit**	6131 (2%)	323 (2%)	1087 (2%)	2251 (3%)
** Other location**	13310 (5%)	1274 (8%)	3297 (7%)	6432 (8%)
**Brain imaging within 1h of arrival**	140797 (54%)	9533 (57%)	26485 (55%)	45096 (55%)
**Swallow screening within 4h of arrival** **(if applicable)**	180621 (69%)	12141 (72%)	32965 (69%)	54844 (66%)
**Stroke unit admission within 4 h of arrival (if applicable)**	147458 (56%)	10874 (64%)	23702 (49%)	33936 (41%)
**Stroke specialist nurse assessment within 24 h of arrival**	237132 (90%)	15389 (91%)	43444 (90%)	74110 (89%)
**Stroke specialist physician assessment within 24 h arrival**	174640 (67%)	14849 (88%)	41878 (87%)	70924 (86%)
**Reperfusion treatment**				
**Intravenous thrombolysis (IVT)**	30489 (12%)	1919 (11.5%)	5020 (10.5%)	8639 (10.5%)
**Of those treated, IVT given within 1 h of hospital arrival**	19026 (62%)	1148 (60%)	3047 (61%)	5224 (60%)
**Mechanical thrombectomy**	3459 (1.5%)	307 (2%)	1062 (2%)	2006 (2.5%)
**Method of assessment by stroke specialist consultant**				
**In person**	158943 (82%)	12673 (78%)	35936 (78%)	61573 (78%)
**Telemedicine (video)**	4550 (2%)	582 (4%)	1862 (4%)	3884 (5%)
**Telephone**	30969 (16%)	2907 (18%)	8076 (18%)	13404 (17%)
**Time to first assessment by stroke nurse, median min [IQR]**	62 [4;244]	50 [1; 214]	50 [1; 249]	47 [1;293]
**Time to first assessment by stroke consultant, median min [IQR]**	321 [58; 1023]	253 [55; 920]	298 [59; 970]	331 [58; 1021]
**Time to Physiotherapy assessment within 72 h, if eligible, median min [IQR]**	1244 [905; 1595]	1182 [838; 1485]	1247 [918; 1599]	1286 [960; 1725]
**Not eligible for physiotherapy assessment**	40501 (15.5%)	2468 (15%)	7487 (15.5%)	14454 (17%)
**Time to Occupational therapy assessment within 72 h, if eligible, median min [IQR]**	1280 [938; 1727}	1220 [877; 1565}	1280 [946; 1715.2]	1318 [986; 1995]
**Not eligible for occupational therapy assessment**	50227 (19%)	3046 (18%)	9604 (20%)	17932 (22%)
**Time to Communication assessment within 72 h, if eligible, median min [IQR]**	1375 [992; 2414}	1311 [930; 2084.5]	1370 [1001; 2390]	1394 [1033; 2431]
**Not eligible for communication assessment**	144108 (55%)	9089 (54%)	25612 (53%)	44708 (54%)
**Time to Swallow assessment within 72 h, if eligible, median min [IQR]**	1198 [437; 1839}	1176 [567; 1650]	1225 [592; 1906}	1199 [516; 1841.5]
**Not eligible for swallow assessment within 72 h**	176023 (67%)	11153 (66%)	31856 (66%)	55049 (66.5%)
**Total length of stay, median days [IQR]**	6.9 [2.6; 20.1]	5.6 [2.2;15.3]	6.7 [2.6;18.8]	6.9 [2.9;20.8]

During the first lockdown, length of acute hospital stay fell by over 1 day compared to pre-pandemic, but this reduction was not seen during the second lockdown, nor maintained post-pandemic. Similar increases were also observed during the first lockdown for receiving a brain scan within 1 hour of hospital arrival and for swallow screening within 4 hours of hospital arrival (Table 2).

**Fig 1:**
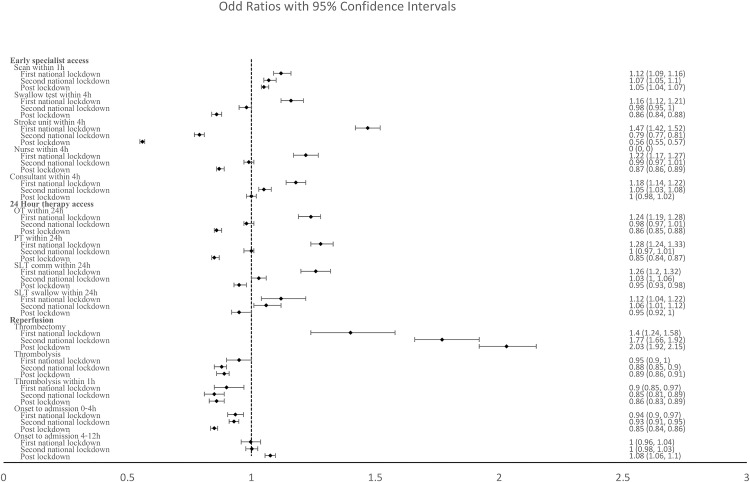
Odds ratios for specialist acute care and treatment across lockdown and post-pandemic periods compared to pre-pandemic baseline.

Fig 1 shows the odds ratios for various aspects of specialist acute care and treatment using logistic regression to compare the respective lockdown and post-pandemic periods to the reference, pre-pandemic period. Time from onset to admission, and the proportions receiving brain scan within 1 hour of arrival and thrombectomy all increased during and post lockdown, but the proportion receiving thrombolysis fell further between the first and second lockdowns. A consistent pattern was observed with many of the quality indicators of access to specialist care and therapy, of an initial improvement in the first lockdown, followed by a deterioration into the second lockdown and post-pandemic periods, along with the sustained lengthening of onset-to-admission (Fig 1). This pattern was most pronounced for stroke unit admission within 4 hours of arrival.

Outcomes were reported for patients at hospital discharge. No substantial changes were observed in the recovery or in-hospital mortality at discharge between the respective analysis periods. However, some changes were observed in the type of discharge destination (Table 3), with an increase in the proportion of patients discharged to a community rehabilitation team, and corresponding falls in the proportions discharged to a care home or the patient’s own home without community rehabilitation.

**Table 3:** Outcomes at discharge.

	Pre-pandemic	First lockdown	Second lockdown	Post pandemic
**Discharge type**				
**Discharged to a care home**	20113 (8%)	1107 (7%)	3033 (6%)	5089 (6%)
**Discharged home**	79004 (30%)	4607 (27%)	12316 (26%)	20751 (25%)
**Discharge somewhere else**	4143 (2%)	236 (1%)	825 (2%)	1289 (2%)
**Transferred to another inpatient care team**	10642 (4%)	607 (4%)	1656 (3%)	2859 (3%)
**Transferred to a community rehabilitation team**	94445 (36%)	7120 (42%)	21021 (44%)	36457 (44%)
**Infection acquired in first 7 days following initial admission**				
**Stroke-associated pneumonia**	21937 (8%)	1601 (10%)	4394 (9%)	6791 (8%)
**Urinary tract infection**	11059 (4%)	630 (4%)	1889 (4%)	2797 (3%)
**Disability at hospital discharge (modified Rankin score)**				
**0**	29143 (11%)	1884 (11%)	4774 (10%)	8088 (10%)
**1**	46951 (18%)	2848 (17%)	7928 (17%)	14460 (17%)
**2**	43465 (17%)	2903 (17%)	8238 (17%)	14667 (18%)
**3**	45491 (17%)	2970 (18%)	9070 (19%)	15546 (19%)
**4**	42258 (16%)	2958 (18%)	8280 (17%)	13432 (16%)
**5**	18549 (7%)	1149 (7%)	3454 (7%)	5648 (7%)
**6 (mortality)**	35494 (14%)	2131 (13%)	6260 (13%)	10891 (13%)
**Good outcome (mRS score ≤2)**	119559 (55%)	7635 (55%)	20940 (54%)	37215 (55%)

**Fig 2:**
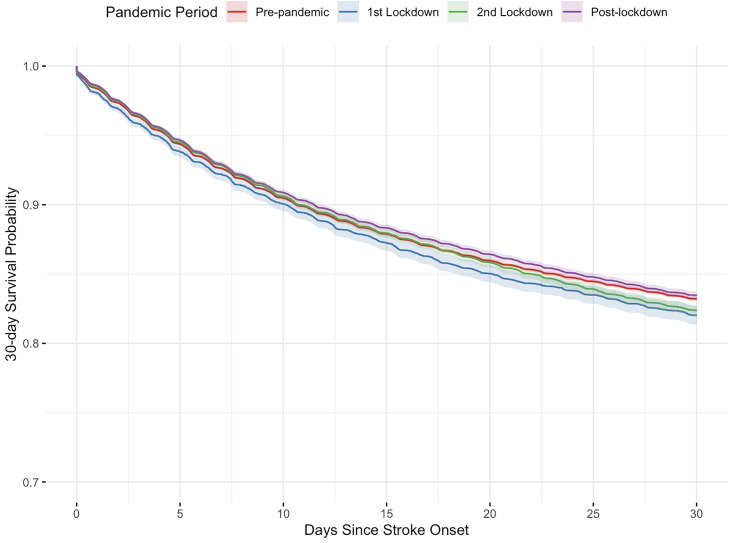
Kaplan-Meier survival curves stratified by the pandemic phases.

The 30-day survival analysis of the first national lockdown showed a decrease in survival time (equivalent to a reduction of −0.2 days; 95% CI: −0.38 to −0.07, p = 0.003) compared to the equivalent pre-pandemic period. This equated to an increase of 7% in the hazard of death during the first lockdown compared to pre-pandemic (HR: 1.07, 95% CI: 1.02 to 1.11, p = 0.002). No differences were observed in mean survival time in the second lockdown period compared to pre-pandemic. In the post-pandemic period, we observed an increase in the 30-day survival time compared to the pre-pandemic period, equivalent to an average increase of 0.1 days (95% CI: 0.03 to 0.17, p = 0.003). This equated to a 3% reduction in the hazard of death within 30 days during the post-lockdown period compared to the pre-pandemic period (HR: 0.97; 95% CI: 0.95 to 0.99, p = 0.003) (Fig 2). Similar patterns were observed for 7-day case-fatality (Table 4).

**Table 4:** Effect of lockdown periods on 7-day and 30-day survival compared to pre-pandemic period.

	First national lockdown		Second national lockdown		Post-pandemic	
	Estimate, 95% CIs	p	Estimate, 95% CIs	p	Estimate, 95% CIs	p
**Average survival time in the first 7 days**	6.7 [6.659-6.698]		6.7 [6.708-6.729]		6.7 [6.718-6.734]	
**7-day Mean difference in survival time**	−0.03 [−0.05--0.01]	0.003	0.01 [−0.00-0.02]	0.113	0.02 [0.01-0.03]	<0.001
**7-day Hazard Ratio**	1.10 [1.04-1.18]	0.002	0.97 [0.93-1.01]	0.117	0.94 [0.91-0.97]	<0.001
**Average survival time in the first 30-day**	26.9[26.346-26.623]		26.7[26.585-26.743]		26.8[26.743-26.861]	
**30-day Mean difference in survival time**	−0.21 [−0.38--0.07]	0.003	−0.04[−0.12-0.05]	0.430	0.10 [0.03-0.17]	0.003
**30-day Hazard Ratio**	1.07 [1.02-1.11]	0.002	1.01 [0.98-1.04]	0.429	0.97 [0.95-0.99]	0.003

## Discussion

Using comprehensive, prospective UK national stroke registry data, we report on the effects of successive ‘waves’ of the COVID-19 pandemic on the delivery and quality of stroke services as well as patient outcomes during the first and second lockdowns of the pandemic in 2020−21, and its subsequent recovery post-pandemic (2021−22). Few nationwide studies have reported on in-hospital stroke care and outcomes longitudinally across the early waves of the pandemic [7,9]. A common observed pattern is that the quality of stroke care for patients admitted to hospital was either maintained or improved during the first pandemic wave compared to the pre-pandemic period. As reported in other studies (1,2), this was explained by increased bed capacity from suspended elective surgery and reduced hospital admissions for stroke with mild deficits, the mobilization of additional staff to clinical areas and the prioritisation of early discharge. Furthermore, the development of regional stroke network models contributed to the delivery of similar or improved rates of key processes to those provided in the pre-pandemic period [10].

Data showed that there were increasing delays from onset to arrival in hospital which were exacerbated throughout the pandemic and have continued to lengthen in the post pandemic period, along with significantly lower rates of patients using ambulance services. These is of concern and requires a systematic approach to address stroke symptom recognition through public health measures, increasing use of pre-hospital validated screening tools and innovations such as pre-hospital video triage [11,12]. Although thrombolysis rates were maintained during the first lockdown, they declined during the second lockdown, and has persisted into the post pandemic period. Likewise, several studies have similarly demonstrated variations in the trajectory of thrombolysis rates across different waves of the pandemic, explained by similar factors as well as additional pressures on workforce [7,13]. A major concern is the failure of thrombolysis rates to recover post-pandemic; this would suggest that there is a sustained effect of delays in onset-to-arrival times post-pandemic still to resolve [14]. By contrast, thrombectomy rates in the UK have continued to increase over time and post pandemic period. The resilience of thrombectomy growth over the pandemic period is contrary to other studies which have demonstrated a reduction in thrombectomy rates, with prolonged door to groin and recanalisation puncture times [15,16]. The concentration of thrombectomy services in a smaller number of regional centers in the UK, with no redeployment of neurointerventional staff during the pandemic, may have relatively protected these services. Additionally, the extension of service operating hours may have facilitated the continued learning and growth of thrombectomy activity into the post pandemic period [17]. Despite some of the improvements in access to evidence-based interventions such as specialist stroke unit care, 7 day and 30 day case fatality rates significantly increased during the first lockdown period as reported previously [3], but then returned to baseline during the second lockdown and subsequently improved in the post-pandemic period. It was not possible to identify whether this was driven by high case fatality rates from stroke patients directly affected with COVID-19 initially as described elsewhere [18] or reduced admissions of milder stroke and other casemix variables during the first lockdown period [3].

Hospital pressures from successive COVID-19 waves in 2020–2021 had a lasting negative impact on specialist acute stroke care quality. Access to stroke units within 4 hours of admission declined sharply after the first lockdown and has not recovered. Despite increased discharge to community rehabilitation, services have struggled to optimise patient flow [19]. Digital health innovations like telerehabilitation and telemedicine were minimally integrated [20]. Flexibility and resilience are key to improving care, as seen in Germany [7], where stroke centers maintained access and reduced case fatality rates by learning and improving with higher vaccination rates.

The strengths of this study are the very large nationwide sample involving high-quality comprehensive data measuring process and outcomes from all hospitals providing acute stroke care, allowing comparisons across the two years of the COVID-19 pandemic and with a matched three-year period beforehand and with the post-pandemic recovery period. The limitations of the study are that a small number of hospitals (n = 9) did not contribute data during the first lockdown period of the pandemic and so it is not possible to know whether the findings would be similar in these non-participating hospitals. Mortality was ascertained by hospital reporting and through linkage to statutory death records from the linked office for natiuonal statistics, but the recording of longer-term disability outcome is not yet comprehensive. COVID-19 status for all patients was not reliably recorded within the SSNAP dataset and would require linkage to other public health testing data in order to examine the association between stroke, case fatality and access to key indicators in this patient population, particularly in ethnically diverse groups.

## Conclusion

The sustained period of acute hospital pressure from recurrent pandemic waves in 2020−21 has had impacted the quality of stroke care and outcomes, specifically the first lockdown. Acute hospital care is still remaining under significant and sustained pressure highlighting the importance of building organizational capacity and resilience, avoiding burnout of clinical staff. An important takeaway from this study is to leverage the insights gained from the disruptions caused by the pandemic to accelerate innovation strategies, such as out-of-hospital care and remote rehabilitation.
